# Increased ERCP-related adverse event from premature urgent ERCP following symptom onset in acute biliary pancreatitis with cholangitis

**DOI:** 10.1038/s41598-024-64644-x

**Published:** 2024-06-13

**Authors:** See Young Lee, Sang Ho Park, Min Young Do, Dong Ki Lee, Sung Ill Jang, Jae Hee Cho

**Affiliations:** 1 Division of Gastroenterology, Department of Internal Medicine, Gangnam Severance Hospital, Yonsei University College of Medicine, 211 Eonjuro, Gangnam-gu, Seoul, 06273 Republic of Korea; 2 Department of Internal Medicine, Yonsei University College of Medicine, Seoul, Republic of Korea; 3 Department of Clinical Research Design and Evaluation, SAIHST, Sungkyunkwan University, Seoul, Republic of Korea

**Keywords:** Choledocholithiasis, Biliary tract disease, Pancreatitis

## Abstract

Acute biliary pancreatitis (ABP) with cholangitis requires endoscopic retrograde cholangiopancreatography (ERCP) within 24 h to resolve ductal obstruction. However, this recommendation is based on the timing of emergency room (ER) visits. We wanted to determine the optimal timing of ERCP for ABP based on the timing of symptom onset, not the timing of the ER visit. We retrospectively reviewed 162 patients with ABP with cholangitis who underwent urgent ERCP (within 24 h of ER admission). Area under the receiver operating characteristic (ROC) curve (AUC) was analyzed to determine differences in complication rates according to time from symptom onset. A difference in ERCP-related adverse events (AEs) was identified, and Youden’s J statistic was used to determine a cutoff time from symptom onset (18 h). We compared mortality and complications based on this cutoff. Based on time to symptom onset, significantly higher rates of aspiration pneumonia (odds ratio [OR] 4.00, 95% confidence interval [CI] 1.15–13.92, *P* = 0.021) and post-ERCP hypotension (OR 11.9, 95% CI 1.39–101.33, *P* = 0.005) were observed in the ≤ 18-h group than in the > 18-h group. The study found that patients who underwent ERCP within 18 h of symptom onset is associated with an increased risk of ERCP-related AEs.

## Introduction

Acute biliary pancreatitis (ABP) occurs when bile duct stones (i.e., gallstones) become stuck near the pancreatic duct opening, resulting in papillary edema. This condition is the primary reason for digestive-disease hospitalizations in the United States, and gallstones are the leading cause of acute pancreatitis (AP)^1^. Patients with ABP are at risk for developing several severe complications, including cholangitis, organ failure, and other life-threatening conditions^2^. Given these possible outcomes, ABP is a medical emergency requiring prompt management to minimize the risk of organ failure. Treatment requires early conservative care with intravenous fluids to alleviate symptoms and decrease disease severity, but ultimately, it is most important to remove the causative gallstones.

Current clinical guidelines for ABP with cholangitis or cholestasis recommend urgent (within 24 h) endoscopic retrograde cholangiopancreatography (ERCP)^3^. However, morbidity and mortality occur in 20–30% of patients receiving urgent ERCP^4–6^, highlighting the need to re-evaluate recommended protocols, including the optimal timing for ERCP.

Following a 1997 study by Folsch et al. that found no clinical benefit for early ERCP performed within 72 h of symptom onset in patients with ABP without obstructive jaundice, most research on optimal ERCP timing has been based on time after emergency room (ER) admission^7^. In this study, we analyzed the optimal timing of ERCP in ABP with cholangitis or cholestasis based on the length of time following symptom onset rather than the time following ER admission to re-evaluate clinical guidelines for urgent ERCP, given that objective data on the exact time of symptom onset are recorded in hospital/clinic electronic medical records (EMRs). We examined the correlations between symptom onset, ERCP timing, and clinical outcomes, specifically focusing on mortality rates and major complications, such as AP-related complications and ERCP-related adverse events (AEs). Through this study aimed at refining our understanding of the optimal timing for ERCP in ABP with cholangitis or cholestasis, we aim to provide valuable insights into how clinical guidelines and practices may be improved to enhance patient management, thereby reducing associated complications.

## Results

### Patient characteristics

**Figure 1:**
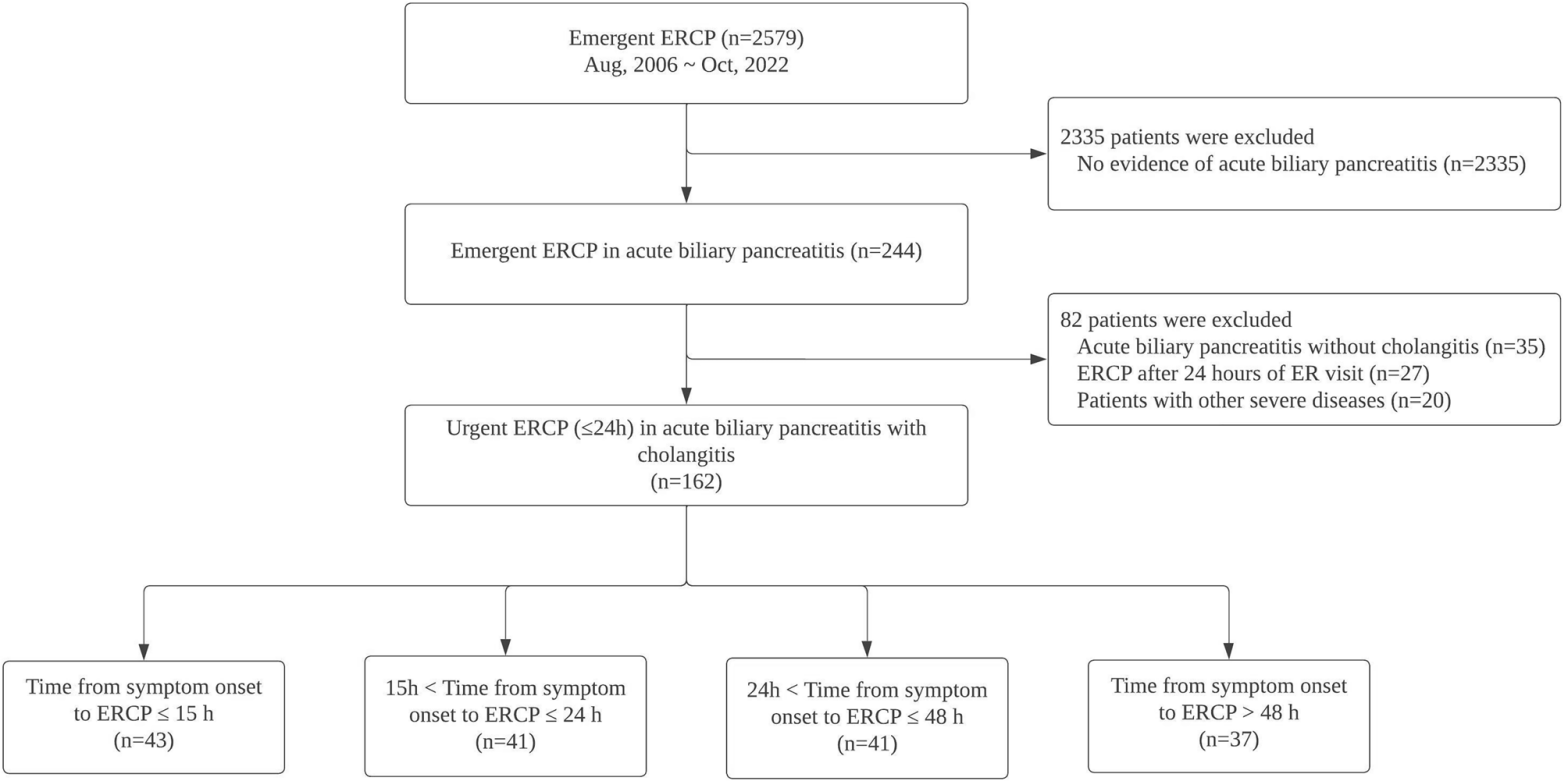
Flow diagram of patient selection according to urgent endoscopic retrograde cholangiopancreatography (ERCP; within 24 h of emergency room admission) in patients with acute biliary pancreatitis with cholangitis.

From August 2006 to October 2022, a total of 2,579 patients presented to our institution’s emergency department and underwent early ERCP (within 72 h of ER admission). Among them, 244 were diagnosed with ABP. Based on the exclusion criteria, we excluded 35 patients who did not meet diagnostic criteria for cholangitis or cholestasis and 27 patients who underwent early ERCP (within 72 h of admission) more than 24 h after admission. Consequently, 162 patients having ABP with cholangitis who underwent urgent ERCP (within 24 h of admission) were included (Fig. 1). The number of patients with mild to moderate AP or severe AP was equal, with 81 total in each category.

The basic characteristics of patients with ABP presenting to the ER and undergoing ERCP are presented (Table 1). Mean age was 60.35 (± 16.79) years, with males representing 48.8% of the cohort. Average body mass index (BMI) was 24.57 (± 3.62). On average, patients visited the ER 34.68 (± 60.62) hours after symptom onset, and ERCP was performed 43.68 (± 61.19) hours after symptom onset. The average time interval between ER admission and ERCP was 8.98 (± 6.60) hours. CT scans performed prior to ERCP revealed APFC in 42.6% of patients. AP-related complications were observed in 14.8% of patients, corresponding to 3.1% with APFC, 8.0% with pseudocyst formation, 1.2% with ANC, and 2.5% with WON.

**Table 1:** Baseline characteristics of patients with acute biliary pancreatitis (ABP) with cholangitis who underwent endoscopic retrograde cholangiopancreatography (ERCP) within 24 h of emergency room (ER) admission.

Baseline characteristics of patient		
	n = 162	%
Age	60.35 ± 16.79	
Male	79	48.8
BMI	24.57 ± 3.62	
HTN	59	36.4
DM	32	19.8
CTSI	1.82 ± 1.61	
Time from symptom onset to ER (hr)	34.68 ± 60.62	
Time from symptom onset to ERCP (hr)	43.68 ± 61.19	
Time from ER visit to ERCP (hr)	8.98 ± 6.60	
Severity of acute pancreatitis†		
Mild AP	81	50.0
Moderate to severe AP	81	50.0
APFC	69	42.6
ERCP-related AEs	18	11.1
Bleeding	2	1.2
Perforation	0	0.0
Duodenitis	1	0.6
Aspiration pneumonia	8	4.9
Post-ERCP hypotension	7	4.3
AP-related complications	24	14.8
APFC	5	3.1
Pseudocyst	13	8.0
ANC	2	1.2
WON	4	2.5

^†^The severity of acute pancreatitis is based on the revised Atlanta classification.

AEs, adverse events; AP, acute pancreatitis; BMI, body mass index; CTSI, CT severity index; ER, emergency room; ERCP, endoscopic retrograde cholangiopancreatography; APFC, acute peripancreatic fluid collection; ANC, acute necrotic collection; WON, walled-off necrosis.

### Cutoff value for primary outcomes for ERCP timing

**Figure 2:**
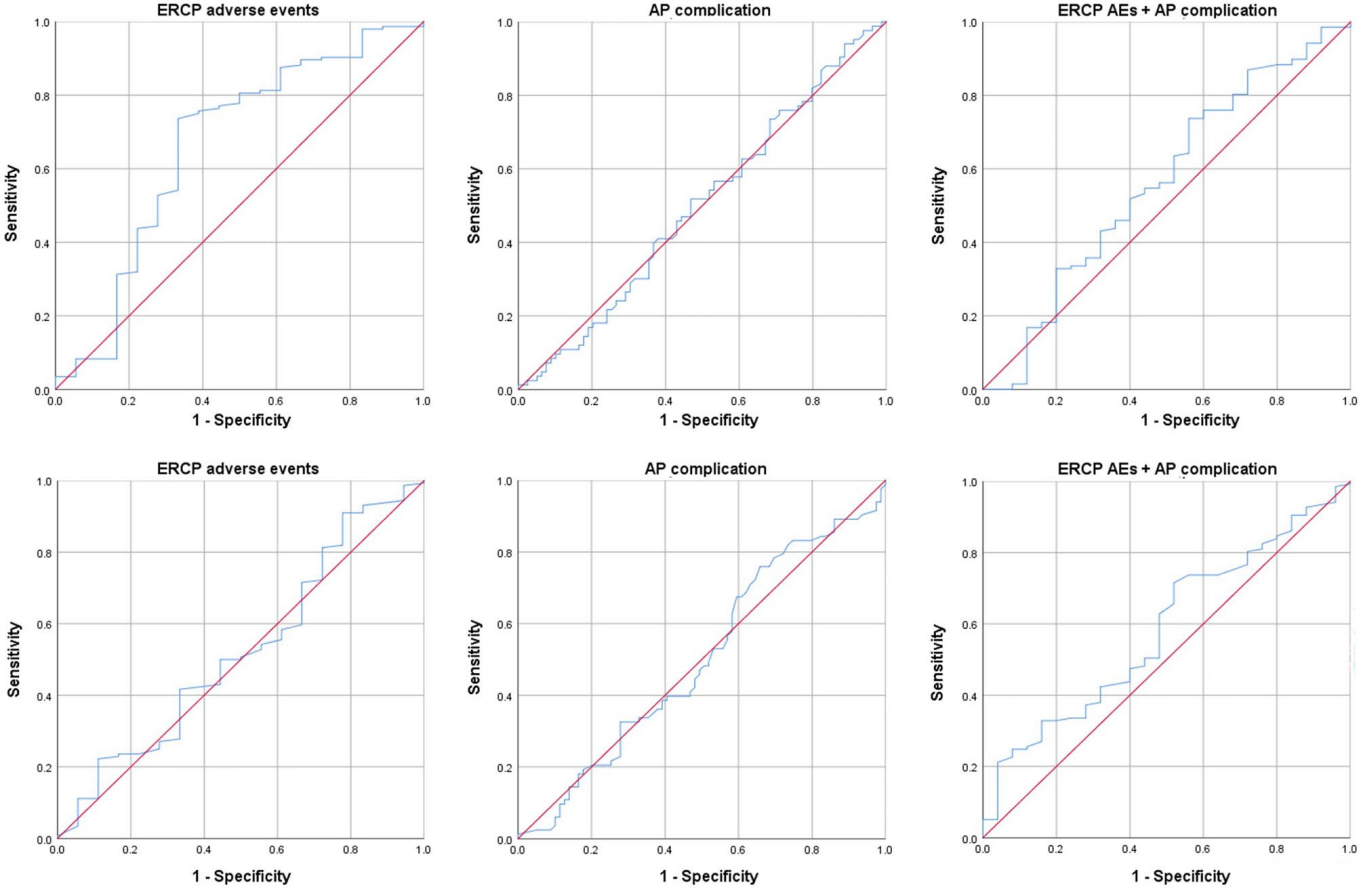
Receiver operating characteristic (ROC) curves of primary outcome relative to time of ERCP. (Top) ROC curves of primary outcome relative to time of ERCP after symptom onset. (Bottom) ROC curves of primary outcome relative to time of ERCP time after ER admission.

We performed ROC curve analyses to determine if there were significant differences in primary outcomes when measured relative to the time from ER admission or to the time from symptom onset in patients who underwent urgent ERCP (within 24 h of ER admission; Fig. 2). For this analysis, we assessed ERCP-related AEs, AP-related complications, and all other complications and detected a significant difference in ERCP-related AEs based on symptom onset time (AUC: 0.662, 95% CI 0.505–0.819; Table 2).

**Table 2:** Results from receiver operating characteristic (ROC) curve analysis of primary outcome relative to timing of ERCP after symptom onset and ER admission.

ROC curve data (primary outcome and symptom onset time)	
Primary outcome	AUC (95% CI)
ERCP-related AEs	0.662 (0.505–0.819)
AP-related complication	0.562 (0.428–0.696)
ERCP-related AEs and AP-related complications	0.551 (0.429–0.672)

ROC curve data (primary outcome and ER visit time)	
Primary outcome	AUC (95% CI)
ERCP-related AEs	0.524 (0.375–0.672)
AP-related complication	0.584 (0.466–0.702)
ERCP-related AEs and AP-related complications	0.523 (0.415–0.630)

AEs, adverse events; AP, acute pancreatitis; AUC, area under the ROC curve; CI, confidence interval; ER, emergency room; ERCP, endoscopic retrograde cholangiopancreatography.

**Figure 3:**
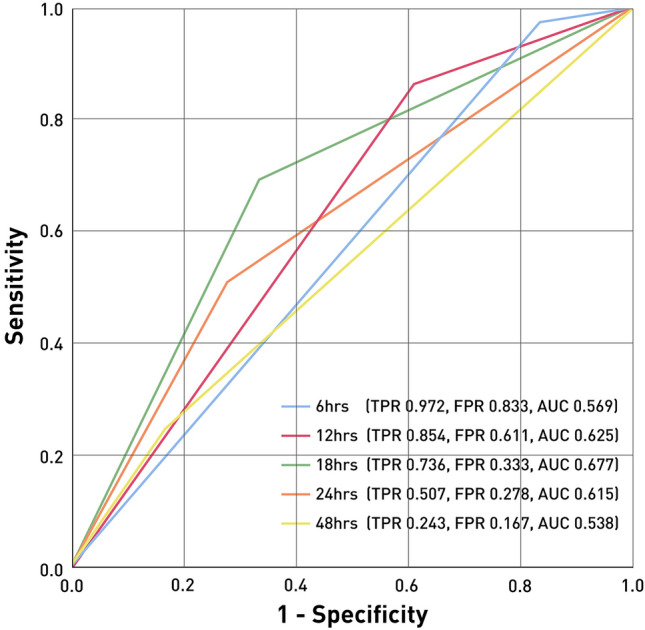
ROC curves of ERCP-related adverse events (AEs) in patients with ERCP performed at various times after symptom onset (i.e., 6 h, 12 h, 18 h, 24 h, 48 h).

We then used Youden’s J statistics to evaluate ERCP-related AEs based on time after symptom onset and identified a cutoff value of 18 h. To confirm this finding, ROC curve values for ERCP-related AEs were calculated at 6, 12, 18, 24, and 48 h from symptom onset. Results show that 18 h is the optimal cutoff, with a true positive rate (TPR) of 0.736, false positive rate (FPR) of 0.333, when divided by 18 h (Fig. 3).

### Comparing baseline characteristics based on symptom onset (≤ 18 h vs. > 18 h)

Based on the above observations, we further compared patients that underwent ERCP within 18 h of symptom onset to those who underwent ERCP after 18 h (Table 3). The ≤ 18-h (n = 58) and > 18-h (n = 104) groups showed no significant differences in age (*P* = 0.558), gender distribution (*P* = 0.855), or BMI (*P* = 0.751). CT severity index scores (P = 0.635), Charlson comorbidity index (CCI) scores (P = 0.512), and severity of AP (P = 0.675) were also comparable between the groups, suggesting similar clinical characteristics, but some laboratory values were slightly different: C-reactive protein (CRP) (26.25 vs. 46.17, *P* = 0.028), hemoglobin (16.45 vs. 13.46, *P* = 0.034), and lipase (3849.8 vs. 3381, *P* = 0.016).

**Table 3:** Baseline characteristics of patients who underwent ERCP stratified based on time of symptom onset.

Baseline characteristics			
	Variable time to ERCP (n = 162)		*P*-value
	≤ 18 h (n = 58)	> 18 h (n = 104)	
Age	59.60 ± 2.18	60.84 ± 1.71	0.558
Male, n (%)	29 (50.0)	50 (48.1)	0.855
BMI	24.32 ± 0.56	24.64 ± 0.45	0.751
HTN, n (%)	19 (32.8)	40 (38.5)	0.471
DM, n (%)	9 (16.7)	23 (22.1)	0.313
Symptom onset to ERCP (hr)	11.33 ± 0.63	63.56 ± 7.04	0.000
WBC	12,394 ± 816	11,817 ± 517	0.592
Hb	16.45 ± 2.56	13.46 ± 0.17	0.034
PLT	221.21 ± 12.68	228.85 ± 7.55	0.662
Hct	49.87 ± 8.68	39.79 ± 0.48	0.051
CRP	26.25 ± 5.70	46.17 ± 6.08	0.028
Total bilirubin	3.02 ± 0.32	3.47 ± 0.27	0.442
Amylase	1286.0 ± 140.2	1092.0 ± 107.4	0.164
Lipase	3849.8 ± 431.5	3381.2 ± 663.3	0.016
CT severity index score	1.26 ± 0.23	1.40 ± 0.16	0.635
CCI	2.26 ± 0.32	2.34 ± 0.19	0.512
Severity of acute pancreatitis†			
Mild AP—n (%)	28 (48.3)	53 (51.0)	0.675
Moderate to severe AP—n (%)	30 (51.7)	51 (49.0)	0.675
ERCP characteristics			
Time from symptom onset to ER (hr)	5.8 ± 3.9	50.8 ± 70.7	< 0.001
Time from symptom onset to ERCP (hr)	12.7 ± 4.2	61.4 ± 70.4	< 0.001
Time from ER visit to ERCP (hr)	7.2 ± 3.5	9.1 ± 5.5	0.121
Residual food in stomach (%)	33.3 ± 7.4	7.6 ± 2.8	< 0.001
Papillary edema (%)	50.0 ± 7.8	31.5 ± 4.9	0.029
Duodenal diverticulum (%)	6.90 ± 3.4	8.65 ± 2.8	0.694
CBD stone or sludge (%)	74.1 ± 5.8	83.7 ± 3.6	0.146
Pancreatic duct cannulation (%)	24.1 ± 5.7	20.2 ± 3.9	0.560
EST (%)	82.8 ± 5.0	90.4 ± 2.9	0.158
Pus (%)	8.62 ± 3.7	6.73 ± 2.5	0.661
ERPD insertion (%)	27.6 ± 5.9	20.2 ± 4.0	0.717
ERBD insertion (%)	41.4 ± 6.5	38.5 ± 4.8	0.284
ERCP procedure time (min)	15.3 ± 1.3	13.6 ± 0.9	0.328
Causes of technical failure			
Duodenal diverticulum—n (%)	1 (1.7%)	4 (3.8%)	0.455
Papillary edema—n (%)	2 (3.4%)	1 (1.0%)	0.262
Technical failure—n (%)	3 (5.2%)	5 (4.8%)	0.918
Technical success—n (%)	55 (94.8%)	99 (95.2%)	0.918

^†^The severity of acute pancreatitis is based on the revised Atlanta classification.

AP, acute pancreatitis; BMI, body mass index; CBD, common bile duct; CCI, Charlson comorbidity index; CRP, C-reactive protein; ERBD endoscopic retrograde biliary drainage; ERCP, endoscopic retrograde cholangiopancreatography; ERPD endoscopic retrograde pancreatic drainage; EST endoscopic biliary sphincterotomy.

Regarding the timing of symptom onset, the interval between symptom onset and ER visit was significantly shorter in the ≤ 18-h group, with a mean of 5.8 h compared to 50.8 h in the > 18-h group. Similarly, the time from symptom onset to ERCP was significantly shorter in the ≤ 18-h group (12.7 h) than in the > 18-h group (61.4 h, *P* < 0.001). However, the difference in time from ER visit to ERCP between the ≤ 18-h group (7.2 h) and the > 18-h group (9.1 h) was not statistically significant (*P* = 0.121).

For ERCP characteristics, technical success rates were similar with no significant difference (94.8% vs. 95.2%, *P* = 0.918), and technical failures were three in the ≤ 18-h group and five in the > 18-h group, with no significant difference in duodenal diverticulum (1.7% vs. 3.8%, *P* = 0.455) and papillary edema (3.4% vs. 1.0%, *P* = 0.262). The mean ERCP procedure time was slightly longer for the ≤ 18-h group (15.3 min) than the > 18-h group (13.6 min), but this difference was not significant (*P* = 0.328). In contrast, the presence of residual food in the stomach was significantly higher in the ≤ 18-h group (33.3 ± 7.4% *vs*. 7.6 ± 2.8%, *P* < 0.001). Incidence of papillary edema was also significantly higher in the ≤ 18-h group (50.0 ± 7.8% *vs*. 31.5 ± 4.9%, *P* = 0.029). However, we observed no significant differences between groups for the remaining ERCP characteristics.

### Comparing primary and secondary outcomes based on symptom onset (≤ 18 h vs. > 18 h)

We next compared primary and secondary outcomes in patients who underwent ERCP ≤ 18 h (n = 58) and > 18 h (n = 104) after symptom onset (Table 4). For ERCP-related AEs, incidence of bleeding was slightly higher in the ≤ 18-h group but not significant (*P* = 0.674). Conversely, occurrence of aspiration pneumonia was higher in the ≤ 18-h group (*P* = 0.021), with a risk ratio of 4.00 (95% CI 1.15–13.92). Post-ERCP hypotension was also significantly higher in the ≤ 18-h group (*P* = 0.005), with a risk ratio of 11.89 (95% CI 1.39–101.33). Moreover, total AEs were significantly higher in the ≤ 18-h group (*P* = 0.004), with a risk ratio of 4.26 (95% CI 1.51–12.06). The groups showed no significant differences in perforation or duodenitis rates (*P* = 1.000 and *P* = 0.453, respectively).

**Table 4:** Comparison of primary and secondary outcomes in patients who underwent ERCP ≤ 18 *vs*. > 18 h after symptom onset.

Primary outcome and secondary outcome				
	Variable time to ERCP (n = 162)		Risk ratio (95% CI)	*P*-value
	≤ 18 h (n = 58)	> 18 h (n = 104)		
ERCP-related AEs				
Bleeding—n (%)	1 (1.72%)	1 (0.97%)	1.81 (0.11–29.44)	0.674
Perforation—n (%)	0 (0.0%)	0 (0.0%)	–	> 0.999
Duodenitis—n (%)	0 (0.0%)	1 (0.97%)	–	0.453
Aspiration pneumonia—n (%)	8 (13.79%)	4 (3.85%)	4.00 (1.15–13.92)	0.021
Post-ERCP hypotension—n (%)	6 (10.34%)	1 (0.97%)	11.89 (1.39–101.33)	0.005
Total adverse events—n (%)	16 (27.59%)	9 (8.65%)	4.26 (1.51–12.06)	0.004
AP-related complication (early)				
APFC—n (%)	24 (41.38%)	45 (43.27%)	0.93 (0.48–1.77)	0.816
ANC—n (%)	1 (1.72%)	3 (2.88%)	0.59 (0.06–5.81)	0.649
AKI—n (%)	6 (10.34%)	6 (5.77%)	1.89 (0.58–6.14)	0.288
Total early complications—n (%)	39 (50.0%)	49 (47.12%)	1.05 (0.55–1.99)	0.811
AP-related complication (late)				
Pseudocyst—n (%)	5 (8.62%)	8 (7.69%)	1.13 (0.35–3.64)	0.835
WON—n (%)	4 (6.90%)	3 (2.88%)	2.49 (0.54–11.55)	0.230
Total late complications—n (%)	10 (17.24%)	9 (8.65%)	2.20 (0.84–5.77)	0.104
AP-related complications (early and late)				
Total AP complications—n (%)	28 (48.28%)	51 (49.04%)	0.97 (0.51–1.84)	0.926
ERCP-related AEs and AP-related complications				
AEs and AP total complications—n (%)	31 (53.45%)	52 (50.0%)	1.15 (0.60–2.19)	0.675
Mortality				
Death—n (%)	1 (1.72%)	1 (0.97%)		0.674
Hospitalization day				
Total length of hospital stay (days)	11.02 ± 1.11	9.00 ± 0.58		0.049

ERCP, endoscopic retrograde cholangiopancreatography; AE, adverse events; AP, acute pancreatitis; APFC, acute peripancreatic fluid collection; ANC, acute necrotic collection; AKI, acute kidney injury; WON, walled-off pancreatic necrosis.

We then assessed early AP-related complications and found that incidence rates of APFC, ANC, and AKI were similar, with risk ratios indicating no significant difference between groups (*P* = 0.816, *P* = 0.649, and* P* = 0.288, respectively). Furthermore, timing of ERCP, whether within or after 18 h, did not significantly impact total early AP-related complications (*P* = 0.811). For late AP-related complications, pseudocyst formation rates were similar, with a risk ratio of 1.13 (95% CI 0.35–3.64; *P* = 0.835). WON rates were higher but not significantly different in the ≤ 18-h group (*P* = 0.230), with a risk ratio of 2.49 (95% CI 0.54–11.55). Similarly, total late AP-related complications were more frequent but not significantly different in the ≤ 18-h group (*P* = 0.104), with a risk ratio of 2.20 (95% CI 0.84–5.77).

Total (both early and late) AP-related complications were comparable between groups, with a risk ratio of 0.97 (95% CI 0.51–1.84) and *P* = 0.926. When considering the combined total of AEs and AP-related complications, there was also no significant difference, with a risk ratio of 1.15 (95% CI 0.60–2.19) and a *P*-value of 0.675. Additionally, there was no difference between groups in mortality (*P* = 0.674), although length of hospitalization stay was significantly longer in the ≤ 18-h group (11.02 ± 1.11 days) than in the > 18-h group (9.00 ± 0.58 days; *P* = 0.049).

## Discussion

In this study, we investigated the association between time from symptom onset prior to ERCP and complication rates in ABP with cholangitis or cholestasis. Our findings suggest that performing urgent ERCP early after symptom onset does not provide a significant benefit for reducing ERCP-related complications, as rates of aspiration pneumonia and post-ERCP hypotension were more common in the ≤ 18-h group than in the > 18-h group. Although this could be misinterpreted as contrary to previous studies, we note that our study evaluated outcomes based on a different measure for ERCP timing. That is, we collected data on urgent ERCP for ABP and re-evaluated clinical outcomes based on the time from symptom onset rather than time from ER admission.

Various studies support early ERCP for pancreatic juice drainage and gallstone removal in patients with ABP, particularly those with biliary obstruction or cholangitis^8–12^. From a pathophysiologic perspective, rapid removal of gallstones can prevent worsening of pancreatitis. However, ABP has a higher rate of ERCP-related AEs than acute cholangitis due to more difficult cannulation and longer procedure times, so caution should be exercised when deciding to perform emergency ERCP^13,14^. In ABP, gallstones may also spontaneously drain into the duodenum as biliary pressure rises, supporting previous findings that indiscriminate emergency ERCP is not optimal. Four randomized controlled trials revealed no benefit from early ERCP (≤ 72 h) in ABP without cholangitis or cholestasis, with some reporting no difference in outcomes compared to conservative treatment without ERCP^15–17^. In addition, some previous studies have reported that early ERCP in ABP is more associated with respiratory-related complications than preceding conservative treatment^7,16^. Between the need for rapid gallstone removal and the risks of performing ERCP in an emergency without adequate preparation, clinicians struggle to decide when to perform ERCP. This dilemma was a primary motivation for our study aimed at investigating primary and secondary outcomes in ABP with cholangitis or cholestasis using a different measure for ERCP timing.

**Figure 4:**
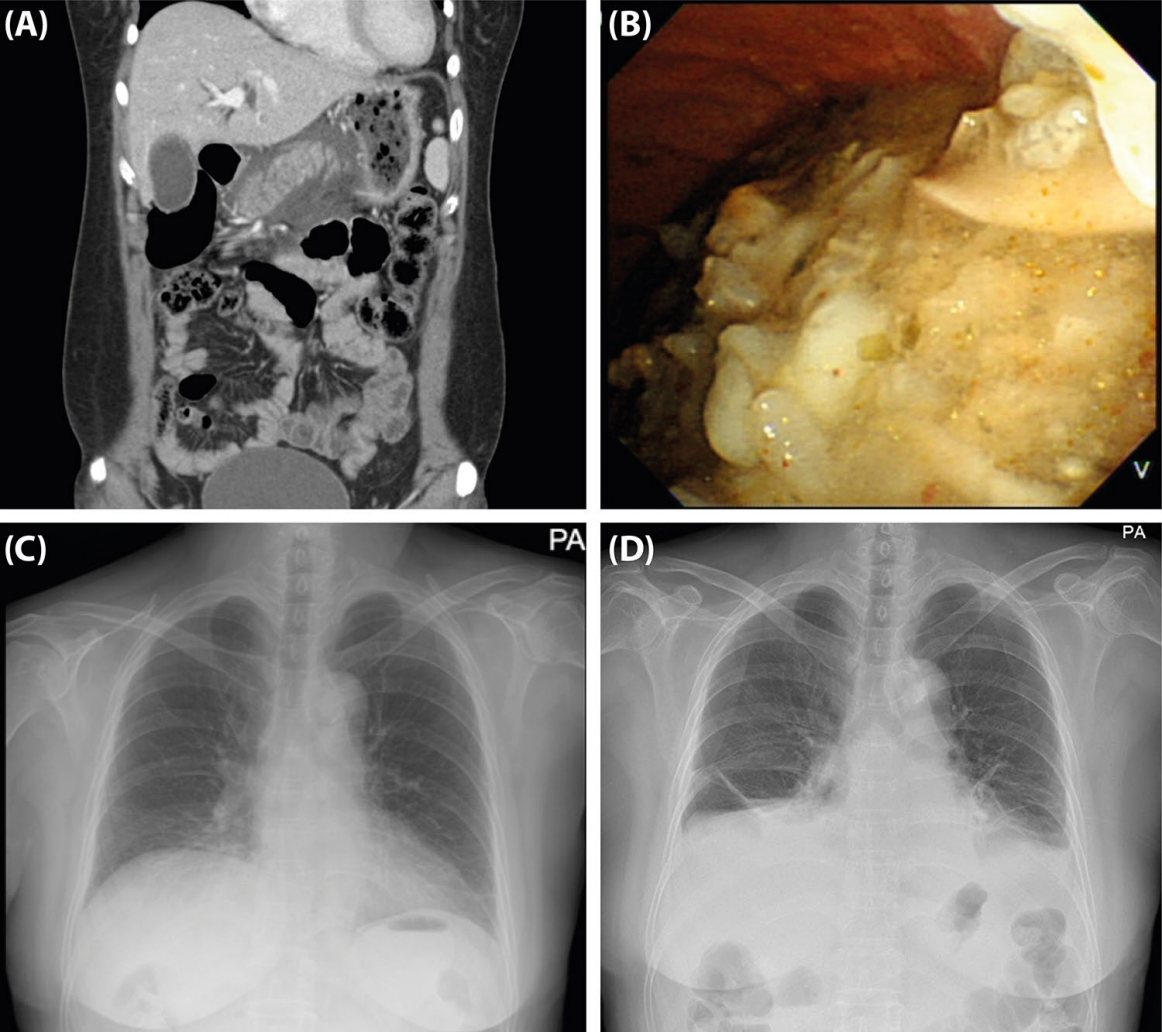
Imaging of patients who underwent ERCP within 18 h of symptom onset. (**A**) Emergency room computed tomography (CT) scan demonstrating peripancreatic fluid collection and edema around the pancreas, as well as residual food in the stomach. (**B**) Large amount of residual food in the stomach during ERCP. (**C**) Chest X-ray taken before ERCP. (**D**) Chest X-ray taken after ERCP, demonstrating pleural effusion in both lower lungs and aspiration pneumonia lesions in the left lower lobe.

There are some differences in laboratory findings in the baseline characteristics between groups (≤ 18 vs. > 18 h), but this may be related to patients presenting to the ER late after symptom onset. Indeed, CRP and lipase, which were higher in the > 18-h group, are known to peak 24 h after the onset of the inflammatory response^18,19^. Although the baseline characteristics of these two groups were similar, we found significant differences in gastric residual food stasis and papillary edema on ERCP. Despite a nothing-by-mouth time of > 8 h prior to ERCP, residual food was identified in the stomachs of 33.3% of the ≤ 18-h group. This finding was likely due to decreased gastrointestinal motility, as AP is associated with impaired intestinal function, such as intestinal dysmotility and ischemia^20–22^. Notably, residual food in the stomach is a significant risk factor for aspiration pneumonia, an ERCP-related AE (Fig. 4) that was more frequently observed in the ≤ 18-h group (13.79% *vs*. 3.88%, risk ratio: 4.00, *P* = 0.021).

We further found that papillary edema, which makes cannulation more difficult during ERCP, occurred more in the ≤ 18-h group. We speculated that this may prolong ERCP times; however, average procedure time was only slightly longer and not significantly different from that in the ≤ 18-h group (15.3 min *vs*. 13.6 min; *P* = 0.328), thus requiring further analysis. Papillary edema poses a challenge for ERCP, so it is reasonable to consider it a risk factor for procedure-related AEs. Indeed, a previous comparative analysis study detected an increased incidence of ERCP-related AEs in patients with difficult ERCP procedures, including longer cannulation and procedure times^23–25^. Post-ERCP hypotension was also more prevalent in the ≤ 18-h group, underscoring the importance of early and adequate fluid resuscitation with intravenous fluids, which is indeed the primary treatment for AP. Urgent ERCP (≤ 24 h) without conservative treatment can cause hemodynamic instability, which can worsen with prolonged ERCP^26,27^.

AP complications, which were categorized into early and late occurrence based on a 4-week demarcation, showed no significant difference between the ≤ 18 h and > 18 h groups. This confirms that regardless of symptom onset timing, there is no significant difference in incidence of AP-related complications if ERCP is performed within 24 h of ER admission.

However, comparative analysis revealed that total hospitalization days, the secondary outcome of this study, was significantly higher in the ≤ 18-h group (*P* = 0.049), a disparity that may be attributed to ERCP-related AEs.

This study has several limitations. First, it utilized a non-randomized retrospective design; the optimal study design would be a prospective randomized controlled trial, which should be considered for future research. Nonetheless, we endeavored to exclude confounding factors related to ERCP timing, including age, BMI, CCI, and baseline blood tests, to mitigate selection bias. Second, this study only includes data from Koreans, so it cannot be said to be representative of people around the world, especially since it is different from Western populations, and further research is needed. Third, the causes of aspiration pneumonia and hypotension in this study could be influenced by various environmental factors during the ERCP procedure, such as CO2 insufflation, the depth of anesthesia, and the precise volume of fluids administered per unit body weight in the ER. A detailed analysis of these variables in future prospective studies would be invaluable in identifying the precise causes of ERCP-related adverse events and in guiding future treatment strategies. Fourth, this was a single-center study with a relatively small, asymmetric sample size of 58 and 104 patients. Therefore, our findings must be validated by increasing the sample size in a multicenter study. Finally, this study did not address the utility of endoscopic ultrasound (EUS). Recent guidelines recommend performing EUS before ERCP, as it can help differentiate cases without gallstones and reduce unnecessary ERCP procedures. It would be interesting to further analyze the utility of EUS combined ERCP in ABP with cholangitis, as EUS may reduce unnecessary adverse events from ERCP.

## Conclusions

This study is the first to analyze ERCPs performed within 24 h based on symptom time after onset. Our findings suggest that even when urgent ERCP (within 24 h) is necessary, performing the procedure too soon (≤ 18 h after symptom onset) may lead to higher rates of aspiration pneumonia and post-ERCP hypotension. Although performing urgent ERCP ≤ 24 h after ER admission for ABP, per existing guidelines, is beneficial for reducing pancreatitis complications, the lack of adequate treatment with antibiotics, analgesics, and intravenous hydration or resuscitation prior to ERCP can complicate the procedure. In particular, residual food in the stomach, papillary edema, and/or patient non-cooperation due to discomfort or pain during the procedure may increase the incidence of ERCP-related AEs.

Based on this study, we suggest that before performing urgent ERCP for ABP with cholangitis or cholestasis, it is important to ascertain the time of symptom onset. If the patient presents early (≤ 18 h after symptom onset), closely assess the patient’s condition, control vital signs, manage pain, provide adequate intravenous fluids, and stabilize the patient before performing ERCP, which may reduce risk of ERCP-related AEs.

## Methods

### Patients

We retrospectively collected clinical data from patients who underwent early ERCP (within 72 h of ER admission) for ABP at Severance Hospital, Gangnam, Korea, from August 2006 to October 2022. Patients with the following characteristics were excluded: (1) absence of concomitant cholangitis or cholestasis; (2) AP not caused by gallstones (no gallstones or sludge identified on cholangiography); (3) ERCP performed more than 24 h after ER admission; and (4) presence of serious underlying medical comorbidities, such as pneumonia, heart failure, or renal failure. We divided patients into groups according to ABP severity (i.e., mild, moderately severe, and severe), and then determined the cutoff value for significant differences in complication rates based on duration of time following symptom onset. The time of symptom onset was defined as the first onset of gastrointestinal symptoms (e.g., abdominal discomfort, abdominal pain) recorded in the EMRs when a patient with a diagnosis of ABP first presented to the ER. Initial laboratory analyses were performed within 6 h of admission, and imaging was performed within the first 24 h of admission. Patients admitted and diagnosed with AP received fluid therapy in the ER following a moderate fluid resuscitation strategy, with an infusion rate of 1.5 ml/kg/hr after an initial bolus of 10 ml/kg for 24 h, unless volume overload was an issue^28^. Confirmed that informed consent was obtained from all participants and/or legal guardians prior to the ERCP. The study was conducted in accordance with the ethical guidelines of the Declaration of Helsinki of 1975 and approved by the Institutional Review Board of Gangnam Severance Hospital (approval number 3-2021-0362).

### ERCP procedure

ERCPs were performed by four skilled endoscopists each possessing more than 5 years of experience and having successfully performed over 1000 ERCPs. During the procedure, patients were maintained under conscious sedation using a combination of propofol, midazolam, and pethidine, while being continuously monitored by an anesthesiologist or endoscopist. All ERCPs were conducted under fluoroscopic guidance to accurately diagnose and manage obstructions, employing a 4.2-mm accessory channel duodenoscope (JF-240, TJF 260 V; Olympus Optical Co., Ltd., Tokyo, Japan). ERCP procedures were generally performed in the prone position; however, in cases where duodenal access was challenging, a lateral position was temporarily adopted until the duodenoscope could be properly positioned. Cannulation of the common bile duct was attempted utilizing a conventional cannula (Contour ERCP Cannula; Boston Scientific, Natick, MA, USA), with or without a guidewire, or alternatively with a pull-type sphincterotome (Clever-cut [Olympus Optical] or Autotome RX 44 [Boston Scientific]). If standard cannulation was ineffective, a precut papillotomy was performed.

### Definitions

AP diagnosis requires that a patient meets at least two of three criteria: (1) abdominal pain characteristic of AP, typically manifesting as sudden, intense epigastric pain, often radiating to the back; (2) serum lipase or amylase activity ≥ 3 times greater than the normal upper limit; and/or (3) distinctive AP features observed in imaging studies such as through use of contrast-enhanced computed tomography or, less frequently, magnetic resonance imaging or transabdominal ultrasonography. AP severity was categorized according to the Revised Atlanta Classification: (1) Mild AP, absence of organ failure and local or systemic complications; (2) Moderately Severe AP, transient organ failure or local or systemic complications resolving within 48 h; and (3) Severe AP, presence of organ failure persisting for more than 48 hours^18,29,30^.

ABP is characterized by the presence of gallstones within the gallbladder or biliary tract. ABP diagnosis requires that a patient meet one or more of the following three criteria: (1) imaging results showing the presence of gallstones or biliary sludge in the gallbladder or biliary tract; (2) imaging results indicating a dilated common bile duct (> 8 mm in diameter for patients younger than 75 years and > 10 mm in diameter for patients aged 75 years or older); and (3) an aminotransferase (ALT/AST) level ≥ 2 times the upper limit of normal^18,30,31^.

Cholangitis diagnosis is based on the presence of fever, cholestasis, and specific imaging findings. Diagnostic criteria for fever require a patient to meet at least one of the following conditions: (1) fever ≥ 38.5 °C with chills, without other apparent cause, or (2) fever ≥ 39 °C without chills, without other apparent cause. Diagnosis of cholestasis requires at least one of the following: (1) jaundice confirmed during physical examination and/or (2) total bilirubin level > 2 mg/dL or elevated levels of AST, ALT, ALP, and gamma-GTP > 1.5 times the upper limit of normal. Imaging findings are confirmed based on the observation of at least one of the following: (1) biliary dilatation and/or (2) strictures, stones, or other underlying causes. This comprehensive diagnostic approach ensures comprehensive, accurate identification of cholangitis, thereby allowing for appropriate treatment and management^16,32^.

Duodenal papillary edema, also known as papillary edema or papillitis, was defined as an unclear demarcation between the papilla and the orad protrusion. The condition is inflammation and swelling of the ampulla of Vater, where the pancreas and bile ducts enter the duodenum. This swelling can obstruct the flow of bile or pancreatic juice, causing symptoms such as jaundice, pain, and complications such as cholangitis or pancreatitis^33,34^.

ERCP-related AEs were defined according to the American Society for Gastrointestinal Endoscopy (ASGE) lexicon, and similar to other studies, only AEs that occurred within 24 h were considered ERCP-related^35,36^. These AEs included: (1) Bleeding, characterized by hematemesis and/or melena or a hemoglobin drop of > 2 g; (2) Perforation, evidence of air or luminal contents detected outside the gastrointestinal tract; (3) Duodenitis, which refers to new-onset duodenal inflammation observed via post-procedural endoscopy; (4) Aspiration pneumonia, indicated by new-onset aspiration pneumonia identified via post-procedural imaging; and (5) Post-ERCP hypotension, defined as a blood pressure reading < 90/50 mmHg or a decrease in blood pressure by 20% or more within 24 h after ERCP.

AP-related complications were classified into early (≤ 4 weeks) and late (> 4 weeks) stages according to the revised Atlanta classification. Early-stage complications included acute peripancreatic fluid collection (APFC), acute necrotic collection (ANC), and acute kidney injury (AKI). Late-stage complications included formation of pseudocysts and walled-off necrosis (WON). If patients presented with APFC prior to ERCP, worsening of APFC detected by post-ERCP CT scans was considered to be a complication^29^. Length of hospital stay was defined as the period spanning from admission to discharge.

### Outcomes

Primary outcomes of this study were AP-related complications and ERCP-related AEs. Secondary outcomes included factors such as duration of hospitalization stay, mortality rates, technical success rates, and clinical success rates. Technical success is defined as the effective removal of stones or sludge from the bile ducts, while clinical success is defined as an uneventful discharge with improved patient symptoms and normalized pancreatic enzyme and inflammatory marker serum levels.

### Statistical analysis

Continuous variables were compared using Mann–Whitney *U,* Kruskal–Wallis, and/or Wilcoxon rank sum tests, whereas categorical variables were analyzed using the Chi-square or Fisher’s exact test, as applicable. Receiver operating characteristic (ROC) curve analysis was performed to assess discriminatory ability of ERCP time for predicting incidence of complications, and Youden’s J statistics were used to determine the optimal cutoff for the number of extended criteria required to identify patients at high risk for complications. ROC analysis results are summarized using the area under the ROC curve (AUC) with a corresponding 95% CI. Logistic regression modeling was used to estimate probability of complications as a function of ERCP time, with 95% CI reported. Statistical analyses were performed using SPSS software, version 23.0 (SPSS Inc., Chicago, IL, USA), and R software, version 4.1.0 (R Foundation for Statistical Computing). *P*-values < 0.05 indicate statistical significance. All data are presented as medians (± standard deviation) or counts and percentages (n [%]), as appropriate.

## Data Availability

The datasets used and/or analyzed in this study are available from the corresponding author upon reasonable request.
